# LncRNA HIF1A-AS2 accelerates malignant phenotypes of renal carcinoma by modulating miR-30a-5p/SOX4 axis as a ceRNA

**DOI:** 10.20892/j.issn.2095-3941.2020.0209

**Published:** 2021-06-15

**Authors:** Mingwei Chen, Xuedong Wei, Xiu Shi, Le Lu, Guangbo Zhang, Yuhua Huang, Jianquan Hou

**Affiliations:** 1 Department of Urology, The First Affiliated Hospital of Soochow University, Suzhou 215006, China; 2 Department of Obstetrics and Gynecology, The First Affiliated Hospital of Soochow University, Suzhou 215006, China; 3 Jiangsu Institute of Clinical Immunology, The First Affiliated Hospital of Soochow University, Jiangsu Key Laboratory of Clinical Immunology, Soochow University, Jiangsu Key Laboratory of Gastrointestinal Tumor Immunology, Suzhou 215006, China

**Keywords:** HIF1A-AS2, SOX4, miR-30a-5p, kidney carcinoma, ceRNA

## Abstract

**Objective:**

Several reports have proposed that lncRNAs, as potential biomarkers, participate in the progression and growth of malignant tumors. HIF1A-AS2 is a novel lncRNA and potential biomarker, involved in the genesis and development of carcinomas. However, the molecular mechanism of HIF1A-AS2 in renal carcinoma is unclear.

**Methods:**

The relative expression levels of HIF1A-AS2 and miR-30a-5p were detected using RT-qPCR in renal carcinoma tissues and cell lines. Using loss-of-function and overexpression, the biological effects of HIF1A-AS2 and miR-30a-5p in kidney carcinoma progression were characterized. Dual luciferase reporter gene analysis and Western blot were used to detect the potential mechanism of HIF1A-AS2 in renal carcinomas.

**Results:**

HIF1A-AS2 was upregulated in kidney carcinoma tissues when compared with para-carcinoma tissues (*P* < 0.05). In addition, tumor size, tumor node mestastasis stage and differentiation were identified as being closely associated with HIF1A-AS2 expression (*P* < 0.05). Knockdown or overexpression of HIF1A-AS2 either restrained or promoted the malignant phenotype and WNT/β-catenin signaling in renal carcinoma cells (*P* < 0.05). MiR-30a-5p was downregulated in renal cancers and partially reversed HIF1A-AS2 functions in malignant renal tumor cells. HIF1A-AS2 acted as a microRNA sponge that actively regulated the relative expression of SOX4 in sponging miR-30a-5p and subsequently increased the malignant phenotypes of renal carcinomas. HIF1A-AS2 showed a carcinogenic effect and miR-30a-5p acted as an antagonist of the anti-oncogene effects in the pathogenesis of renal carcinomas.

**Conclusions:**

The HIF1A-AS2-miR-30a-5p-SOX4 axis was associated with the malignant progression and development of renal carcinoma. The relative expression of HIF1A-AS2 was negatively correlated with the expression of miR-30a-5p, and was closely correlated with SOX4 mRNA levels in renal cancers.

## Introduction

Malignant tumors greatly affect human health^1,2^. Renal cell carcinoma (RCC) accounts for 2%–3% of adult malignant tumors, and the prognoses of patients with advanced kidney carcinomas is very poor, especially for those individuals with distant metastasis^3^. The treatment of renal malignancies therefore represents an ongoing challenge. Various studies have reported possible mechanisms and causes of kidney carcinomas^4–11^; however, the precise mechanism of RCC remains unclear.

The lncRNA is a novel kind of noncoding RNA, which is at least 200 nucleotides in length. The lncRNAs may participate in the progression of various diseases, especially malignant tumors^12–17^. The lncRNAs, as important tumor gene regulators, effect tumor biological behavior, transcriptional regulation, and post-transcriptional regulation^18–22^. Evidence suggests that lncRNAs play vital roles in malignant carcinomas, and have recently been reported to contribute to migration, proliferation, apoptosis, metastasis, and other biological processes^23–28^.

The lncRNA, HIF1A-AS2, has been shown to be involved in the progression of various malignancies^29–41^. For example, HIF1A-AS2 exacerbates the growth of gastric cancer^29^, while it accelerates triple-negative breast carcinoma cell invasion and proliferation, and has been shown to enhance paclitaxel resistance^30–33^. Abnormal expression of HIF1A-AS2 has also been reported to be involved in the progression of bladder cancer^34,35^. The expression of HIF1A-AS2 in mesenchymal glioblastoma is related to hypoxia and sponging to miR-153-3p, which mediates HIF-1α expression and accelerates angiogenesis from hypoxia in human umbilical vein endothelial cells (HUVECs)^36^. HIF1A-AS2 sponges miR-129-5p to regulate DNMT3A expression and the epithelial-mesenchymal transition (EMT) in colorectal cancer^37^. The HIF1A-AS2/miR-665/IL6 system also regulates osteogenic differentiation^38^. HIF1A-AS2 regulates HIF-1A expression through transcriptional competition involving adenosine deaminase acting on RNA^39^. Downregulated HIF1A-AS2 also affects the pathogenesis and progression of preeclampsia^40^. HIF1A-AS2 sponges miR-33b-5p to regulate SIRT6 in osteosarcomas^41^. Our study was therefore directed toward investigating the potential role of HIF1A-AS2 in the progression of carcinomas, as well as assessing its contribution to the progression and development of renal carcinoma. Similar to most malignancies, there are some therapeutic challenges related to renal cancers, which have existed for a long period of time.

The microRNAs (miRNAs) are 18–25 nucleotide non-coding RNAs, which can regulate the tumorigenesis and progression of many tumors^42–49^. The miRNAs also play a role in the evolution of species, and miR-30a-5p has been reported to play a role in many diseases, including cholangiocarcinoma^42^; it can also act as a negative regulator, contributing to the evolution of renal carcinomas.

Extensive studies have reported that lncRNAs function as miRNAs sponges^50–56^. We first reported a mutual correlation between HIF1A-AS2 and miR-30a-5p as miRNA sponges in renal carcinomas. These findings increased our knowledge of the expression patterns of lncRNA-miRNA sponges.

In the present study, HIF1A-AS2 was shown to be highly expressed in renal cancer tissues and cells, with miR-30a-5p being present only in small amounts. HIF1A-AS2 expression was closely correlated with differentiation across tumor node metastasis (TNM) stages. HIF1A-AS2 was found to facilitate renal cancer progression, while miR-30a-5p suppressed this process. Mechanistically, the upregulated expression of HIF1A-AS2 may inhibit the relative expression of miR-30a-5p, to subsequently increase the expression of SOX4 at a posttranscriptional level. Furthermore, HIF1A-AS2 functioned in a ceRNA-dependent manner to sponge miR-30a-5p to tightly regulate SOX4 expression. HIF1A-AS2 also acted as a significant tumor regulator and potential therapeutic target. The HIF1A-AS2-miR-30a-5p-SOX4 axis was involved in the progression of renal carcinomas, which highlights its possible application in clinical diagnosis and therapy.

## Materials and methods

### Patient samples

Our study included kidney carcinoma patients who received tumorectomy. We quick-froze the kidney carcinoma tissues and paired normal peritumoral specimens in liquid nitrogen after resection. We received written informed consent from each patient. Our experimental protocol was approved by the Institutional Ethics Review Board of the First Affiliated Hospital of Soochow University (Approval No. 2019110).

### Cell lines and cell culture

The cells were cultured in an incubator at 37 °C and 5% CO_2_. The 786-O, ACHN, OS-RC-2, and 293T cell lines were obtained from the Institute of Cell Biology, Chinese Academy of Sciences, Shanghai, China. A total of 1% antibiotics (100 U/mL penicillin and 100 μg/mL streptomycin sulfate) and 10% fetal bovine serum (FBS) were added into Minimal Essential Medium (MEM), Dulbecco’s Modified Eagle Medium (DMEM), and RPMI 1640. The 786-O, OS-RC-2, ACHN, and 293T cells were cultivated in RPMI 1640, MEM, or DMEM.

### Cell transfection

Specific siRNA oligonucleotides were transiently transfected into renal cancer cells using si-HIF1A-AS2 sense (5’-GAGUUGGAGGUGUUGAAGCAAAUAU-3’) and antisense (5’-AUAUUUGCUUCAACACCUCCAACUC-3’). Si-NC and si-RNA (si-HIF1A-AS2) were purchased from Gene Pharma (Suzhou, China). SOX4-specific siRNAs (si-SOX4, sc-38412) were purchased from Santa Cruz Biotechnology (Santa Cruz, CA, USA) and Gene Pharma (Suzhou, China). The renal carcinoma cells were cultivated in 6-well plates and transfected using Lipofectamine 3000 Transfection Reagent according to the manufacturer’s instructions (Thermo Fisher Scientific, Waltham, MA, USA). The plasmid vectors (pcDNA3.1-HIF1A-AS2, and the negative control) were purchased from Gene Pharma. The cells were cultured for at least 24 h before transfection, and the transfected cells with the corresponding vector were collected after 48 h of transfection.

### RT-PCR

Total RNA was extracted from the specimens and renal cells using TRIzol reagent (Invitrogen, Carlsbad, CA, USA) based on the product description. The cDNA was synthesized from whole RNA using the Prime Script RT Reagent Kit with gDNA Eraser (Takara, Dalian, China). SYBR Premix Ex Taq II (Takara) was used to detect the relative expression levels of HIF1A-AS2 using RT-qPCR and the CFX96 sequence detection system (Bio-Rad, Hercules, CA, USA). **Supplementary Table S1** shows the main primer sequences. The endogenous controls were glyceraldehyde 3-phosphate dehydrogenase (GAPDH) and U6 small nuclear RNA. A relative quantification method (*2^-^*^ΔΔ*Ct*^) was used to calculate the expressions, which were normalized to endogenous controls.

### Cell proliferation assays

Cell proliferation was detected using the CCK-8 assay (Beyotime, Shanghai) based on the product description. SiRNAs or plasmids were used to transfect cells, which had been incubated for 24 h in 96-well plates. A microplate reader (Bio-Rad) was used to measure the absorbance in each well at 0, 24, 48 and 72 h after transfection.

### The 5-ethyl-2′-deoxyuridine (EdU) incorporation assay

An EdU Apollo DNA *in vitro* kit (Ribobio, Guangzhou, China) used the EdU incorporation assay based on the product descriptions. Briefly, cells transfected with siRNA or plasmid were incubated for 2 h at 37 °C, then treated with 100 μL of 50 μM EdU per well, followed by fluorescence microscopy to visualize the cells.

### Cell migration assay

The cells were transferred to 6-well plates, and were cultured in an incubator until 90%–100% confluent, followed by siRNA or plasma transfection of the cells. A 200 μL sterilized pipet tip was then used to generate clean lines in 6-well plates. Cell images were then captured using a digital camera. After 24 h, the images of cells were again captured using a digital camera.

### Flow cytometry assay

SiRNAs or plasmid vectors were respectively transfected in kidney carcinoma cells. After 48 h of transfection, cells were collected and resuspended in fixation fluid, which included 5 μL annexin V-FITC, 10 μL propidium iodide, and 195 μL cell suspension. Flow cytometry (Beckman Coulter, San Jose, CA, USA) was used to detect cell apoptosis.

### Western blot analysis

Total protein was separated by 10% SDS–PAGE and transferred to polyvinylidene difluoride membranes. After blocking in 5% nonfat milk, the membranes were incubated overnight for 16 h in 4 °C with the primary antibody. The membranes were then incubated for 1–2 h with a secondary antibody, and an enhanced chemiluminescence ECL kit (Beyotime, Shanghai, China) was used to visualize the bands. β-Actin, tubulin, or GAPDH were used as internal standards. The antibodies used are listed in **Supplementary Table S2**.

### Luciferase reporter assays

TCF (T cell factor) transcription factor activity was used to measure canonical Wnt signaling pathway activity. TOP or FOP flash and Renilla-luciferase plasmids were used to transfect renal cells. The luciferase activity was analyzed using a DLR assay system (Promega, Madison, WI, USA). PmirGLO Dual-luciferase vectors (Fubio, Shanghai, China) were used to clone the binding and mutant sequences. HIF1A-AS2 or SOX4 wild type (WT) or mutant type (MUT) was constructed and co-transfected along with miR-30a-5p mimics or normal control (NC), then transfected with Lipofectamine 3000 and incubated for 48 h. A microplate reader was used to measure the luciferase activities.

### Animal experiments

The 5-week-old male BALB/c nude mice were divided into 2 groups, with each group comprised of 6 mice. LV-NC and LV-HIF1A-AS2 were made by Gene Pharma (Shanghai). A total of 2 × 10^6^ OS-RC-2 cells were injected into the mouse dorsal flank regions, and tumor growth was measured every 5 days. The formula, a × b^2^/2 (*a*: long diameter; b: short diameter) was used to calculate tumor volume. Finally, mice were sacrificed after 30 days, and each subcutaneous tumor was weighed. The animal experimental protocol was approved by the Ethics Committee of Soochow University (Approval No. ECSU-20190002018).

### RNA fluorescence *in situ* hybridization (FISH)

For FISH analyses, renal cells were immobilized in 4% formaldehyde, treated with pepsin, and dehydrated with ethanol. The 786-O, ACHN, OS-RC-2, and 293T cells were incubated in hybridization buffer with FISH probes using HIF1A-AS2 (Robbio, Guangzhou, China) for 24 h. After hybridization, the slides were rinsed and dehydrated. The slides were then mounted using Prolong^®^ Gold Antifade Reagent (Thermo Fisher Scientific) using 4′,6-diamidino-2-phenylindole for detection, followed by confocal laser scanning microscopy (Zeiss, Jena, Germany).

### Statistical analysis

Every experimental assay was conducted at least in triplicate. The data are presented as the mean ± standard deviation (SD). Statistical analyses were conducted using SPSS statistical software for Windows, version 20.0 (SPSS, Chicago, IL, USA). The relative expression analyses of HIF1A-AS2 were conducted using the paired sample *t*-test. Analysis of variance was used to analyze the CCK-8 assay data. Other data analyses used the independent samples *t*-test. *P* < 0.05 was regarded as statistically significant.

## Results

### Upregulated expression of HIF1A-AS2 and downregulated expression of miR-30a-5p in kidney carcinomas

**Figure 1:**
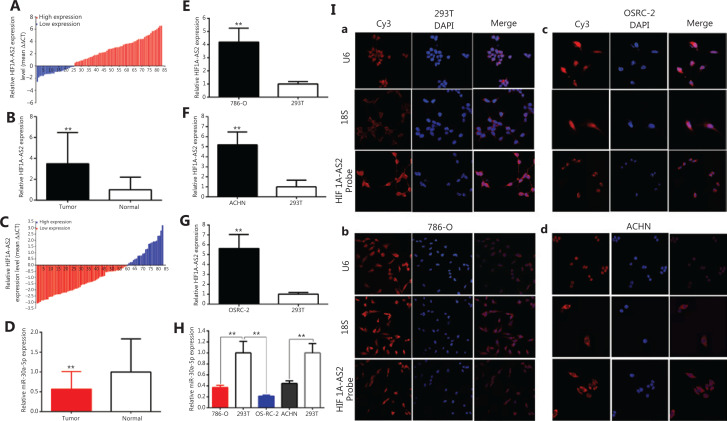
The relative expressions of HIF1A-AS2 and miR-30a-5p in renal carcinoma patients’ samples and cells. Samples were detected, and the distribution of HIF1A-AS2 was mainly in the cytoplasm. *GAPDH* was the internal control gene. The relative expression patterns of HIF1A-AS2 (A and B) and miR-30a-5p (C and D) are shown in paired kidney carcinoma tissues and normal tissues. We also compared their expression levels in renal carcinoma cells (786-O, ACHN, and OS-RC-2 cells) and 293T cells (F-H). The distribution of HIF1A-AS2 was analyzed by fluorescence *in situ* hybridization in 786-O, ACHN and OS-RC-2, and 293T cells [I (a-d)]. The18S RNA and U6 indicated cytoplasm and nucleus, respectively. (^*^P < 0.05; ^**^P < 0.01).

Compared to para-carcinoma specimens, the relative expression of HIF1A-AS2 was increased in 69.88% (58 of 83) carcinoma specimens (*P* < 0.001) by approximately 3.472-fold (**Figure 1A and 1B**), with the relative expression of miR-30a-5p decreasing in 71.08% (59 of 83) of carcinoma samples (*P* < 0.01) (**Figure 1C and 1D**). Compared to 293T cells, the relative expression of HIF1A-AS2 was significantly upregulated in kidney carcinoma cells, 786-O (approximately 4.200-fold, *P* < 0.01), ACHN cells (approximately 5.184-fold, *P* < 0.01), and OS-RC-2 cells (approximately 1.998-fold, *P* < 0.001) (**Figure 1E–1G**). The relative expressions of miR-30a-5p were decreased in renal carcinoma cells, with 786-O approximately 62.79% (*P* < 0.01), OS-RC-2 approximately 78.83% (*P* < 0.01), and ACHN approximately 55.70% (*P* < 0.01) (**Figure 1H**). High expression of HIF1A-AS2 was closely associated with tumor size (*P* < 0.05), differentiation (*P* < 0.01), and TNM stages (*P* < 0.01) (**Table 1**). However, there was no correlation between HIF1A-AS2 and sex, age, and lymph node metastasis. Follow-up animal studies indicated that HIF1A-AS2 promoted tumorigenicity *in vivo* (**Figure 7A–7F**). These results showed that HIF1A-AS2 acted as a tumor enhancer, and that miR-30a-5p acted as an anti-oncogene in the kidney carcinomas.

**Table 1:** Correlation between HIF1A-AS2 expression and clinicopathological characteristics of renal cell cancer patients (clear cell renal cell carcinomas)

Characteristics	Total	Expression of HIF1A-AS2		*P*
		Higher, *n* = 58	Lower,* n* = 25	
Gender				0.326
Male	51	38 (74.5%)	13 (25.5%)	
Female	32	20 (62.5%)	12 (37.5%)	
Age (years)				0.456
≤ 50	28	18 (64.3%)	10 (35.7%)	
> 50	55	40 (72.7%)	15 (27.3%)	
Tumor size (cm)				0.027*
≤ 7	31	17 (54.8%)	14 (45.2%)	
> 7	52	41 (78.8%)	11 (21.2%)	
Differentiation				0.007**
Moderate/poor	49	40 (81.6%)	9 (18.4%)	
Well	34	18 (52.9%)	16 (47.1%)	
TNM stage				0.007**
T0-1	31	16 (51.6%)	15 (48.4%)	
T2-4	52	42 (80.8%)	10 (19.2%)	
Lymph node metastasis				0.548
N0	67	48 (71.6%)	19 (28.4%)	
N1 or above	16	10 (62.50%)	6 (37.5%)	

^*^P < 0.05; ^**^P < 0.01. Tumor node metastasis according to staging TNM of the American Joint Committee on Cancer in 2010.

### LncRNAHIF1A-AS2 was mainly distributed in the cytoplasm

The cellular localizations of lncRNAs were used to assess possible functions and then revealed their potential mechanisms of action, including chromatin remodeling and translational regulation. Using the fractionation indicators of 18S RNA and U6, FISH was used to show that HIF1A-AS2 was mainly localized to the cytoplasm of kidney cell lines [**Figure 1I (a–d)**].

### Knockdown of HIF1A-AS2 suppressed the cell proliferation of kidney cell lines, and the upregulation of HIF1A-AS2 promoted cell proliferation of renal cell lines

**Figure 2:**
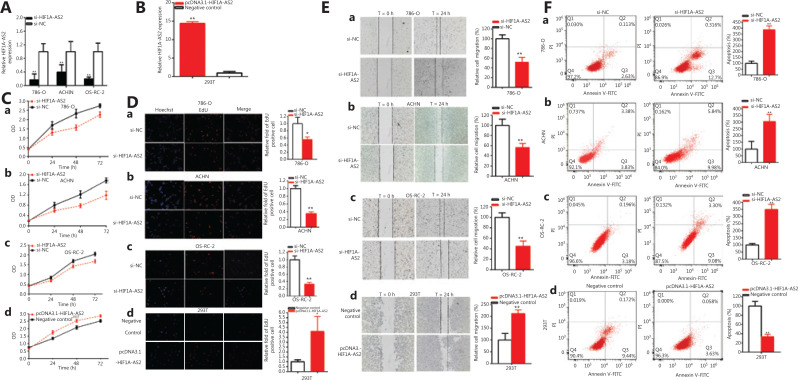
Knockdown or overexpression of HIF1A-AS2 suppressed or promoted cell progression. The relative expression level of HIF1A-AS2 was significantly downregulated by si-HIF1A-AS2 (A) and pcDNA3.1-HIF1A-AS2 (B). Analysis of variance was used for the comparison of curves of cell proliferation. Cell proliferation was detected in both renal carcinoma cells after transfection of si-RNA [C (a-c)] and pcDNA3.1-HIF1A-AS2 [C (d)]. Representative images of EdU assays and the relative fold changes of EdU positive cells were detected by si-RNA [D (a–c)] and pcDNA3.1-HIF1A-AS2 [D (d)]. The relative cell migration was suppressed after transfection of si-RNA, and the representative images were as follow [E (a–c)]. The relative cell migration was promoted after transfection of pcDNA3.1-HIF1A-AS2, and the representative images are shown in [E (d)]. Apoptotic cells were measured after transfection of si-RNA [F (a–c)] and pcDNA3.1 [F (d)] by flow cytometry analyses. (^*^P < 0.05; ^**^P < 0.01).

The relative expression of HIF1A-AS2 was detected by qRT-PCR at 48 h after transfection of siRNAs in 786-O, ACHN, and OS-RC-2 cells, and after transfection of pcDNA3.1-HIF1A-AS2 into 293T cells. The relative expression levels of HIF1A-AS2 were downregulated by 83.04% in 786-O (*P* < 0.001), downregulated by 60.30% in ACHN (*P* < 0.01), and decreased by 80.17% in OS-RC-2 cells (*P* < 0.001). The relative expression levels of HIF1A-AS2 were significantly decreased by si-HIF1A-AS2 after 48 h of transfection (**Figure 2A**), and the relative expression levels of HIF1A-AS2 were prominently increased by 14.43-fold in 293T cells (*P* < 0.001) at 48 h after transfection of pcDNA3.1-HIF1A-AS2 (**Figure 2B**).

CCK-8 assays detected whether si-HIF1A-AS2 restrained cell proliferation in 786-O, ACHN and OS-RC-2 cell lines and whether pcDNA3.1-HIF1A-AS2 promoted the proliferation in 293T renal cells. The data showed that knockdown of HIF1A-AS2 [**Figure 2C (a–c)**] significantly restrained cell proliferation in kidney carcinoma cells (*P* < 0.01 in renal cell lines). PcDNA3.1-HIF1A-AS2 [**Figure 2C (d)**] in 293T cells remarkably accelerated cell proliferation (*P* < 0.01).

Cell proliferation was also detected using EdU assays. As shown [**Figure 2D (a–d)**], compared to the control group, EdU positive 786-O, ACHN, and OS-RC-2 cells in the knockdown of HIF1A-AS2 groups were decreased, while the pcDNA3.1-HIF1A-AS2 groups showed the reverse results.

The EdU assay showed that the EdU positive cell quantities in the si-HIF1A-AS2 group were decreased by 44.15% in 786-O cells (*P* < 0.05) [**Figure 2D (a)**], reduced by 64.41% in ACHN cells (*P* < 0.001) [**Figure 2D (b)**], and reduced by 66.81% in OS-RC-2 cells (*P* < 0.001) [**Figure 2D (c)**]. The Edu positive cell quantities were increased by 4.110-fold in 293T cells (*P* < 0.05) [**Figure 2D (d)**] in the pcDNA3.1-HIF1A-AS2 group.

Together, the results showed that downregulation of HIF1A-AS2 decreased cell proliferation in renal cell lines, and upregulation of HIF1A-AS2 facilitated cell proliferation in renal cell lines.

### Downregulation of HIF1A-AS2 decreased cell migration of kidney cell lines, and overexpression of HIF1A-AS2 promoted kidney cell line migration

Cells were transfected with siRNA and plasmids in 6-well plates, and cell scratch assays were used to detect the roles of siRNA and plasmids in cell migration. The scratch assay showed that the ratio of the relative migration of the si-HIF1A-AS2 group was decreased by 48.38% in 786-O cells (*P* < 0.01) [**Figure 2E (a)**], 43.57% in ACHN cells (*P* < 0.01) [**Figure 2E (b)**], and 54.80% in OS-RC-2 cells (*P* < 0.01) [**Figure 2E (c)**]. The ratio of the relative migration of the pcDNA3.1-HIF1A-AS2 group was upregulated 2.118-fold in 293T cells (*P* < 0.01) [**Figure 2E (d)**]. The results demonstrated that downregulation of HIF1A-AS2 decreased renal cell migration, while upregulation of HIF1A-AS2 promoted renal cell migration.

### Silencing HIF1A-AS2 enhanced renal cell apoptosis, and upregulation of HIF1A-AS2 suppressed renal cell apoptosis

Knockdown or overexpression of HIF1A-AS2 may regulate renal cell apoptosis after transfection of plasmids and siRNA. Flow cytometry was used to detect cell apoptosis. The apoptotic renal cells were observably upregulated after transfection with si-HIF1A-AS2. Compared to the si-NC groups, the ratios of apoptosis were significantly upregulated by 3.859-fold in 786-O cells (*P* < 0.001), 3.053-fold in ACHN cells (*P* < 0.01), and 3.502-fold in OS-RC-2 cells (*P* < 0.001) [**Figure 2F (a–c)**] after transfection with HIF1A-AS2 knockdown.

Compared to the negative control groups, the ratios of apoptosis were downregulated by 66.22% in 293T cells (*P* < 0.001) [**Figure 2F (d)**] after transfection with pcDNA3.1-HIF1A-AS2. Overall, knockdown of HIF1A-AS2 accelerated renal cell apoptosis, and upregulation of HIF1A-AS2 suppressed renal cell apoptosis.

### Upregulated miR-30a-5p inhibited renal carcinoma cell proliferation and migration, and promoted renal carcinoma cell apoptosis

**Figure 3:**
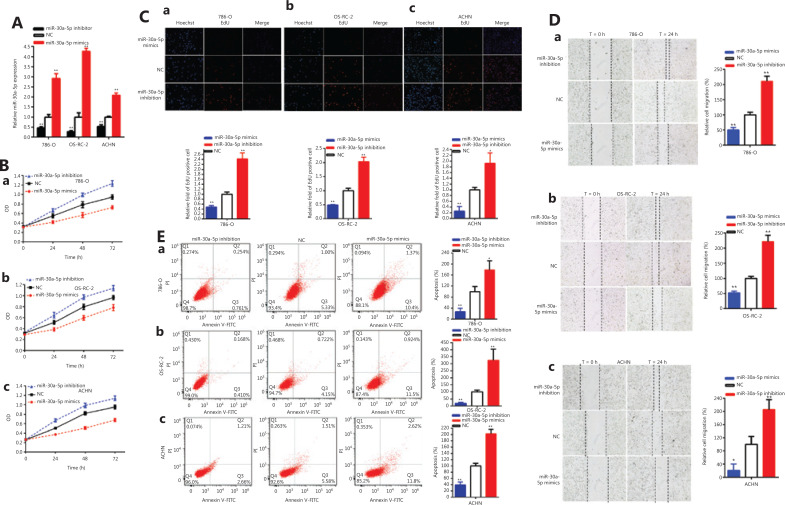
The miR-30a-5p acted as an anti-oncogene. The relative expression level of miR-30a-5p was significantly downregulated by the miR-30a-5p inhibitor and increased by miR-30a-5p mimics (A). We detected cell proliferation after miR-30a-5p inhibitor and miR-30a-5p mimic transfection [B (a-c)] in renal carcinomas cells. Representative images of EdU assays and the relative fold changes of EdU positive cells were detected by the miR-30a-5p inhibitor and miR-30a-5p mimics [C (a–c)]. The relative cell migration was inhibited or accelerated after miR-30a-5p mimics or inhibitor transfection in renal cancer [D (a–c)] cell lines. Flow cytometry analysis was used to measured apoptotic cells after miR-30a-5p mimics or inhibitor transfection in the renal cancer [E (a–c)] cell lines.(^*^P < 0.05; ^**^P < 0.01).

The relative expression levels of miR-30a-5p were significantly reduced by 54.28% in 786-O cells (*P* < 0.01), 72.66% in OS-RC-2 cells (*P* < 0.01), and 47.42% in ACHN cells (*P* < 0.01) at 48 h after transfection with an miR-30a-5p inhibitor (**Figure 3A**). The relative expression levels of miR-30a-5p were increased approximately 2.916-fold in 786-O cells (*P* < 0.001), 4.265-fold in OS-RC-2 cells (*P* < 0.001), and 2.084 times in 786-O cells (*P* < 0.001) at 48 h after transfection with miR-30a-5p mimics (**Figure 3A**).

The CCK-8 data showed that miR-30a-5p mimics decreased renal carcinoma cell proliferation, while the miR-30a-5p inhibitor accelerated renal carcinoma cell proliferation [**Figure 3B(a–c)**] (*P* < 0.01).

The quantity of Edu-positive cells in the miR-30a-5p mimic group was decreased by 51.82% in 786-O cells (*P* < 0.001), 51.10% in OS-RC-2 cells (*P* < 0.01), and 74.13% in ACHN cells (*P* < 0.01) [**Figure 3C (a–c)**]. The level of Edu positive cells in the miR-30a-5p inhibitor group was upregulated approximately 2.421-fold in 786-O cells (*P* < 0.001), 2.032-fold in OS-RC-2 cells (*P* < 0.001), and 1.926-fold (*P* < 0.05) in ACHN cells [**Figure 3C (a–c)**]. Our study showed that upregulation of miR-30a-5p decreased renal carcinoma cell proliferation and suppression of miR-30a-5p accelerated renal carcinoma cell proliferation.

The ratios of relative migrations were downregulated 49.83% in 786-O cells (*P* < 0.01), 47.68% in OS-RC-2 cells (*P* < 0.001), and 78.98% in ACHN cells (*P* < 0.05) [**Figure 3D (a–c)**] after transfection with miR-30a-5p mimics. The ratio of the relative migration was upregulated 2.108-fold in 786-O cells (*P* < 0.001), 2.212-fold in OS-RC-2 cells (*P* < 0.001), and 2.053-fold in ACHN cells (*P* < 0.01) [**Figure 3D (a–c)**] after transfection with the miR-30a-5p inhibitor. These results showed that higher miR-30a-5p expression suppressed renal carcinoma cell migration and decreased miR-30a-5p accelerated renal carcinoma cell migration.

Compared with the NC groups, the ratios of apoptosis were increased by 1.786-fold in 786-O cells (*P* < 0.05), 3.246-fold in OS-RC-2 cells (*P* < 0.01), and 2.022-fold in ACHN cells (*P* < 0.001) [**Figure 3E (a–c)**] after transfection with miR-30a-5p mimics. Compared to the NC groups, the ratios of apoptosis were decreased by 72.91% in 786-O cells (*P* < 0.01), 81.07% in OS-RC-2 cells (*P* < 0.001), and 60.73% in ACHN cells (*P* < 0.01) [**Figure 3E (a–c)**] after transfection with the miR-30a-5p inhibitor. Overall, increased miR-30a-5p expression increased renal carcinoma cell apoptosis and reduced miR-30a-5p decreased renal carcinoma cell apoptosis.

### HIF1A-AS2 sponged miR-30a-5p

**Figure 4:**
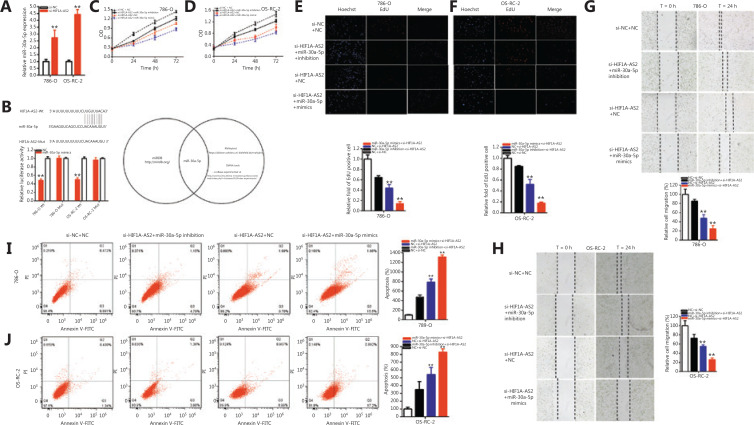
HIF1A-AS2 was a target of miR-30a-5p. The relative expression of miR-30a-5p was increased by si-HIF1A-AS2 (A). Dual-luciferase reporter assays were performed in 786-O and OS-RC-2 cells co-transfected with HIF1A-AS2-Wt or HIF1A-AS2-Mut and miR-30a-5p mimics or normal control (NC) (B). We detected cell proliferation after co-transfection with si-NC+NC in both renal carcinoma cell lines, si-HIF1A-AS2+miR-30a-5p inhibitor or si-HIF1A-AS2+miR-30a-5p mimics by CCK-8 and Edu assays (C-F). The relative cell migration after co-transfection with si-NC+NC, si-HIF1A-AS2+miR-30a-5p inhibitor or si-HIF1A-AS2+miR-30a-5p mimics, and the representative images are shown (G and H). The apoptotic cells were measured by flow cytometry analysis after co-transfection with si-NC+NC, si-HIF1A-AS2+miR-30a-5p inhibitor or si-HIF1A-AS2+miR-30a-5p mimics (I and J). (^*^P < 0.05; ^**^P < 0.01).

Compared to the NC groups, the relative expressions of miR-30a-5p were increased 2.727-fold in 786-O cells (*P* < 0.01) and 4.427-fold in OS-RC-2 cells (*P* < 0.001) (**Figure 4A**) in the si-HIF1A-AS2 groups. **Figure 4B** shows that bioinformatics databases were used to predict binding sites of HIF1A-AS2 and miR-30a-5p. The predictions were confirmed using the luciferase reporter assay. In addition, miR-30a-5p mimics suppressed HIF1A-AS2 wild-type reporter luciferase activity; when compared to the co-transfections using NC+ pmirGLO-HIF1A-AS2-Wt, the luciferase activity was reduced 51.44% in 786-O cells (*P* < 0.001) and 49.42% in OS-RC-2 cells (*P* < 0.001) in the co-transfections miR-30a-5p mimics + pmirGLO-HIF1A-AS2-Wt groups, however, miR-30a-5p did not suppress the HIF1A-AS2 mutant reporter vector luciferase activity (**Figure 4B**). Overall, the luciferase reporter assays verified that HIF1A-AS2 sponged miR-30a-5p.

### HIF1A-AS2 sponging miR-30a-5p mediated renal carcinoma cell progression

First, we verified that miR-30a-5p co-regulated progression induced by HIF1A-AS2 in renal carcinomas cells. The si-HIF1A-AS2 co-transfected miR-30a-5p mimics better suppressed renal carcinoma cell proliferation (**Figure 4C–4F**) and migration (**Figure 4G and 4H**) than the si-NC co-transfection with NC (si-NC+NC), and compared with the si-NC+NC group, apoptosis was increased in the si-HIF1A-AS2 co-transfection miR-30a-5p mimics (si-HIF1A-AS2+miR-30a-5p) group (**Figure 4I and 4J**). In contrast, the miR-30a-5p inhibitor partially reversed the inhibition effects on renal carcinoma cell progression induced by si-HIF1A-AS2.

The CCK-8 assays showed that si-HIF1A-AS2 co-transfected miR-30a-5p mimics (**Figure 4C and 4D**) decreased renal carcinoma cell proliferation in both 786-O and OS-RC-2 cells (all, *P* < 0.01). In addition, the miR-30a-5p inhibitor partially reversed the decreased effects on renal carcinoma cell proliferation induced by si-HIF1A-AS2 (**Figure 4C and 4D**).

Compared to si-NC+NC, si-HIF1A-AS2 co-transfected miR-30a-5p mimics decreased the quantity of Edu positive cells by 85.99% in 786-O cells (*P* < 0.001) and 82.23% in OS-RC-2 cells (*P* < 0.001). In addition, the miR-30a-5p inhibitor partially reversed the inhibitory effects on renal carcinoma cell quantity of Edu positive cells induced by si-HIF1A-AS2, and increased it by 20.68% in 786-O cells and 32.87% in OS-RC-2 cells (**Figure 4E and 4F**).

Compared to si-NC+NC, si-HIF1A-AS2 co-transfected miR-30a-5p mimics decreased the ratio of the relative migrations by 74.80% in 786-O cells (*P* < 0.001) and 73.91% in OS-RC-2 cells (*P* < 0.001). Moreover, the miR-30a-5p inhibitor partially reversed the inhibitory effects on renal carcinoma cell migration induced by si-HIF1A-AS2, and increased it by 37.20% in 786-O cells and 17.78% in OS-RC-2 cells (**Figure 4G and 4H**).

Compared to si-NC+NC, si-HIF1A-AS2 co-transfected miR-30a-5p mimics increased the ratio of relative apoptosis by 13.08-fold in 786-O cells (*P* < 0.001) and 8.292-fold in OS-RC-2 cells (*P* < 0.001). Furthermore, the miR-30a-5p inhibitor partially reversed the increased apoptosis in renal carcinoma cells induced by si-HIF1A-AS2 and reduced it by 315.6% in 786-O cells and 192.4% in OS-RC-2 cells (**Figure 4I and 4J**).

### HIF1A-AS2 sponging miR-30a-5p closely regulated SOX4

**Figure 5:**
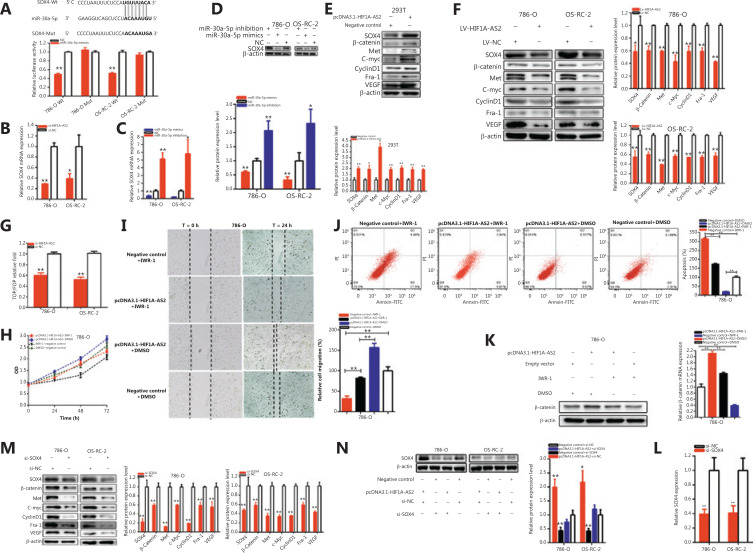
HIF1A-AS2 positively regulates SOX4 expression via sponging miR-30a-5p. SOX4-Wt and miR-30a-5p mimics co-transfection inhibited luciferase activity (A) as shown using the dual-luciferase reporter assay. The relative expression level of SOX4 was decreased by si-HIF1A-AS2 (B). The relative expression level of SOX4 was regulated by miR-30a-5p mimics and inhibition (C). Overexpressing miR-30a-5p downregulated SOX4 expression and knockdown of miR-30a-5p upregulated SOX4 expression in renal carcinomas cells (D). Overexpression of HIF1A-AS2 upregulated SOX4 expression in 293T cells (E). Knockdown of HIF1A-AS2 downregulated SOX4 expression in renal carcinomas cells (F). Knockdown of HIF1A-AS2 downregulated transcriptional activity of the Wnt/β-catenin pathway as determined by the TOP flash/FOP flash reporter luciferase activity assay (G). CCK-8 assay (H), migration (I), flow cytometric analysis (J), Western blot and qPCR (K) showed that IWR-1 inhibited and even reversed the effect of pcDNA3.1-HIF1A-AS2 on cell progression in 786-O cells. Knockdown of SOX4 downregulated β-catenin expression in renal carcinomas cells (M). Knockdown of SOX4 reversed malignant phenotypic promotion of renal carcinomas cells induced by HIF1A-AS2. Si-SOX4 reversed SOX4 expression promotion induced by overexpression of HIF1A-AS2 in renal carcinomas cells (N). The relative expression level of SOX4 was decreased by si-SOX4 (L). (^*^P < 0.05; ^**^P < 0.01).

We used bioinformatics databases to predict mutual binding sites of SOX4 and miR-30a-5p, which is shown in **Figure 5A**. The predicted binding sites and binding effects were determined using the luciferase reporter assay.

Compared to co-transfections with NC + pmirGLO-HIF1A-AS2-Wt, our results confirmed that miR-30a-5p mimics dramatically decreased SOX4 wild-type reporter luciferase activity, which was decreased to 50.43% in 786-O cells (*P* < 0.001) and 48.45% in OS-RC-2 cells (*P* < 0.001) in the co-transfection with miR-30a-5p mimics + SOX4-3’-UTR-Wt. In contrast, miR-30a-5p could not decrease the SOX4 mutant binding site reporter vector luciferase activity (**Figure 5A**).

We confirmed whether HIF1A-AS2 regulated SOX4 expression in a miR-30a-5p-dependent manner in renal carcinoma cells (**Figure 5B–5D**). Compared to the si-NC groups, the SOX4 expressions were decreased by 70.92% in 786-O cells (*P* < 0.001) and 60.93% in OS-RC-2 cells (*P* < 0.05) (**Figure 5B**) in the si-HIF1A-AS2 groups. Our study showed that the relative expression of HIF1A-AS2 was closely related to SOX4 expression, and that downregulated HIF1A-AS2 reduced SOX4 expression in renal carcinomas cells. Furthermore, compared to the si-NC groups, the relative expression levels of SOX4 were decreased by 63.09% in 786-O cells (*P* < 0.01) and 76.90% in OS-RC-2 cells (*P* < 0.001) (**Figure 5C**) in the miR-30a-5p mimic groups, and the relative expression levels of SOX4 were increased 5.150-fold in 786-O cells (*P* < 0.01) and 5.821-fold in OS-RC-2 cells (*P* < 0.05) (**Figure 5C**) in the miR-30a-5p inhibition groups. Our studies suggested that overexpression of miR-30a-5p reduced SOX4 expression and miR-30a-5p inhibition upregulated SOX4 expression in renal carcinomas cells (**Figure 5D**). Together, our results showed that HIF1A-AS2 closely regulated SOX4 expression via sponging miR-30a-5p in renal carcinoma cells.

Our further experiments confirmed that decreased HIF1A-AS2 could alter WNT signaling in renal carcinomas cells (**Figure 5E and 5F**). Western blot was used to detect the expression of WNT signaling-associated downstream genes. Overexpression of HIF1A-AS2 increased SOX4, MET, C-myc, cyclinD1, Fra-1, VEGF, and β-catenin expressions in renal cells (**Figure 5E**); knockdown of HIF1A-AS2 decreased SOX4, MET, C-myc, cyclinD1, Fra-1, VEGF, and β-catenin expression in renal carcinomas cells (**Figure 5F**).

TOP/FOP flash is a method for the determination of intracellular beta-catenin-mediated transcription activity. TOP/FOP flash reporter assays were used to detect whether HIF1A-AS2 functioned via the WNT/β-catenin signaling pathway. Data illustrated that decreasing HIF1A-AS2 expression downregulated Wnt-activity in the 786-O and OS-RC-2 cells (**Figure 5G**).

IWR-1 (a β-catenin inhibitor, IC_50_ = 180 nM, sc-295215, Santa Cruz), which is a tyrosine kinase inhibitor, suppressed the Wnt/β-catenin signaling pathway. To further verify whether HIF1A-AS2 was involved in the Wnt signaling pathway we used IWR-1. IWR-1 was involved in the inhibitory effects of si-HIF1A-AS2 in kidney carcinoma cells. Cells that were continuously transfected with pcDNA3.1-HIF1A-AS2 and co-incubated with IWR-1 showed stronger inhibitory effects on cell proliferation (**Figure 5H**) and migration (**Figure 5I**) of kidney carcinoma cells, with comparison to the pcDNA3.1-HIF1-AS2+DMSO group. Apoptosis (**Figure 5J**) was remarkably increased in the pcDNA3.1-HIF1AS2 co-transfected IWR-1 group compared with the pcDNA3.1-HIF1-AS2+DMSO group in 786-O cells. In contrast, the IWR-1 inhibitor partially reversed the tumorigenic effects induced by HIF1A-AS2. Our further experiments showed that IWR-1 dramatically reversed the promotion of β-catenin expression induced by overexpressing HIF1A-AS2 in renal carcinomas cells (**Figure 5K**).

### Silencing of SOX4 reversed the malignant renal carcinoma cell phenotype promotion of HIF1A-AS2 overexpression

We wished to confirm whether HIF1A-AS2 regulated malignant phenotypes in a SOX4-dependent manner in renal carcinomas cells.

**Figure 6:**
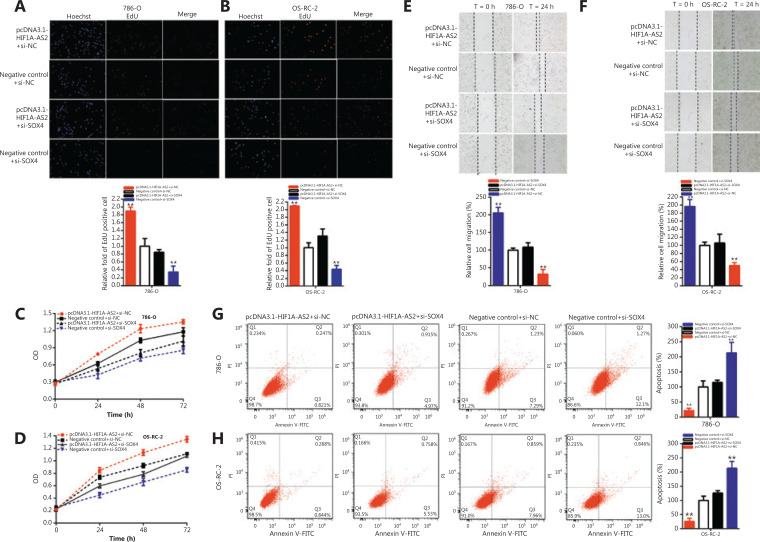
HIF1A-AS2 positively regulates SOX4 expression via sponging miR-30a-5p. Knockdown SOX4 reversed cell proliferation promotion induced by overexpression of HIF1A-AS2 (EdU, A and B; CCK8, C and D). Knockdown of SOX4 reversed cell migration promotion induced by overexpression of HIF1A-AS2 (E and F). Knockdown SOX4 reversed cell apoptosis inhibition induced by overexpression of HIF1A-AS2 (G and H). (^*^P < 0.05; ^**^P < 0.01).

Further experiments confirmed that decreasing SOX4 could alter WNT signaling in renal carcinomas cells. Knockdown of SOX4 decreased MET, C-myc, cyclinD1, Fra-1, VEGF, and β-catenin expressions in renal carcinomas cells (**Figure 5M**). Further experiments showed that the knockdown of SOX4 dramatically reversed the promotion of SOX4 expression induced by overexpressing HIF1A-AS2 in renal carcinoma cells (**Figure 5N**). The relative expression levels of SOX4 were reduced by 60.53% in 786-O cells (*P* < 0.01) and 59.09% in OS-RC-2 cells (*P* < 0.01) at 48 h after transfection of si-SOX4 (**Figure 5L**). Moreover, knockdown of SOX4 reversed the promotion of renal carcinoma cell proliferation (**Figure 6A–6D**) induced by overexpression of HIF1A-AS2. Furthermore, SOX4 knockdown reversed renal carcinoma cell migration (**Figure 6E and 6F**) induced by overexpression of HIF1A-AS2. In addition, SOX4 knockdown reversed renal carcinoma cell apoptosis suppression (**Figure 6G and 6H**) induced by overexpression of HIF1A-AS2.

Compared to negative control+si-NC, pcDNA3.1-HIF1A-AS2 co-transfected si-NC increased the quantity of Edu positive cells by 1.892-fold in 786-O cells (*P* < 0.01) and 2.09-fold in OS-RC-2 cells (*P* < 0.001), and si-SOX4 significantly decreased the quantity of Edu positive cells by 65.33% in 786-O cells (*P* < 0.05) and 56.34% in OS-RC-2 cells (*P* < 0.01). Moreover, si-SOX4 partially reversed its stimulatory effects on renal carcinoma cells, and the quantity of Edu positive cells induced by pcDNA3.1-HIF1A-AS2 decreased by 104.5% in 786-O cells and 78.93% in OS-RC-2 cells (**Figure 6A and 6B**).

The CCK-8 assay results showed that pcDNA3.1-HIF1A-AS2 co-transfected si-NC accelerated renal carcinoma cell proliferation, and si-SOX4 co-transfected si-NC was reversed (*P* < 0.01) in 786-O and OS-RC-2 cells. Si-SOX4 partially reversed the stimulatory effects on renal carcinoma cell proliferation induced by pcDNA3.1-HIF1A-AS2 (**Figure 6C and 6D**).

Compared to the negative control+si-NC, pcDNA3.1-HIF1A-AS2 co-transfected si-NC increased the ratio of the relative migration 2.053-fold in 786-O cells (*P* < 0.001) and 1.967-fold in OS-RC-2 cells (*P* < 0.001), and si-SOX4 co-transfected si-NC significantly decreased the ratio of the relative migration to 67.34% in 786-O cells (*P* < 0.01) and 50.27% in OS-RC-2 cells (*P* < 0.01). Moreover, si-SOX4 partially reversed the stimulatory effects on renal carcinoma cell migration induced by pcDNA3.1-HIF1A-AS2 and decreased by 97.08% in 786-O cells and 91.12% in OS-RC-2 cells (**Figure 6E and 6F**).

Compared to the negative control+si-NC, pcDNA3.1-HIF1A-AS2 co-transfected si-NC decreased the ratio of relative apoptosis 78.16% in 786-O cells (*P* < 0.01) and 74.04% in OS-RC-2 cells (*P* < 0.01), and si-SOX4 co-transfected si-NC significantly increased the ratio of the relative apoptosis by 2.132-fold in 786-O cells (*P* < 0.01) and 2.133-fold in OS-RC-2 cells (*P* < 0.01). Furthermore, si-SOX4 partially reversed the repression of apoptosis in renal carcinoma cells induced by pcDNA3.1-HIF1A-AS2 and increased by 93.60% in 786-O cells and 99.59% in OS-RC-2 cells (**Figure 6G and 6H**).

Together, our results indicated that HIF1A-AS2 accelerated malignant renal carcinoma cell phenotypes in a SOX4-dependent manner.

### Knockdown of HIF1A-AS2 decreased tumorigenicity of kidney carcinoma cells

**Figure 7:**
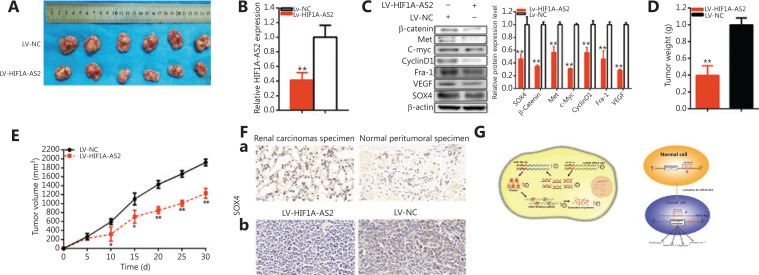
The HIF1A-AS2 effect on renal carcinoma cell tumorigenicity. Tumors collected from mice are shown (A). The relative expression level of HIF1A-AS2 was decreased by LV-HIF1A-AS2 (B). Knockdown of HIF1A-AS2 downregulated SOX4. (C). The tumor weights of LV-HIF1A-AS2 or LV-NC treatment groups were measured and analyzed (D). We measured and analyzed the tumor volumes of the LV-HIF1A-AS2 or LV-NC treatment groups (E). Knockdown of HIF1A-AS2 downregulated SOX4 expression in renal cancer tissue and *in vivo* renal carcinoma cells [F(a and b)]. The schematic diagram of the oncogenic role of HIF1A-AS2 in renal carcinoma cells (G). HIF1A-AS2 functions as a miRNA sponge to positively regulate SOX4 expression by sponging miR-30a-5p and subsequently promoting malignant phenotypes of renal carcinoma cells, thus playing an oncogenic role in renal carcinomas pathogenesis.(^*^P < 0.05; ^**^P < 0.01).

Xenograft models were further used to determine whether HIF1A-AS2 regulated tumorigenicity of kidney carcinoma cells. We found that knockdown of HIF1A-AS2 decreased the tumorigenicity of kidney carcinoma cells *in vivo* (**Figure 7A–7F**). Tumors collected from mice are shown and measured (**Figure 7A**). Downregulation of HIF1A-AS2 expression was significant when compared to the control group of kidney carcinoma cells* in vivo* (**Figure 7B**). We found that LV-HIF1A-AS2 downregulated SOX4, β-catenin, Met, C-myc, cyclinD1, Fra-1, VEGF, and the expression of kidney carcinoma cells *in vivo* (**Figure 7C**). Compared to the LV-NC treatment group, the tumor weights were less in the LV-HIF1A-AS2 group (**Figure 7D**). Tumor growth of the LV-NC treatment group was faster than that in the LV-HIF1A-AS2 group (**Figure 7E**). Immunohistochemistry assays showed that the relative protein expression level of HIF1A-AS2 was upregulated in the renal cancer tissues, and that the knockdown of HIF1A-AS2 downregulated SOX4 expression *in vivo* in renal carcinoma cells [**Figure 7F(a, b)]**. These data showed that HIF1A-AS2 facilitated tumorigenicity of kidney carcinoma cells *in vivo*.

As shown in **Figure 7G**, HIF1A-AS2 was significantly upregulated in renal carcinoma cells and HIF1A-AS2 sponged miR-30a-5p to closely regulate SOX4 expression. Upregulated SOX4 protein facilitated transcription and translation of proteins operating through abnormal protein signaling pathways, and subsequently accelerating malignant renal carcinoma phenotypes.

## Discussion

Studies have reported that lncRNAs are abnormally expressed RNAs with more than 200 nucleotides, which play special roles in various diseases, especially malignant tumor formation^12–28^. As key components in regulating gene expression and tumor progression, studies of lncRNAs have expanded our understanding of their biological behavior during diseases, especially during carcinoma^18–28^. In addition, reports have shown that the lncRNAs are significant biomarkers and possible treatment targets.

LncRNA HIF1A-AS2 is located on chromosome 14, NC_000014.9, and has been found to be overexpressed, to act as an oncogene in many tumor tissues, including gastric carcinoma^29^, triple-negative breast carcinoma^30–33^, bladder carcinoma^34,35^, glioblastoma multiforme^36^ and osteosarcoma^38^. HIF1A-AS2 is also involved in the progression of diseases in tissues, such as human umbilical vein endothelial cells, colorectal cancer, adipose used to extract stem cells, coronary artery disease, and preeclampsia^37,39,40^. In most cases, HIF1A-AS2 is involved in the progression and tumorigenesis of carcinomas and functions as an oncogene. Knowledge of the basic structures and interactions of lncRNAs with other cellular biomolecules can provide direction for further research to reveal the mechanism of tumorigenesis and tumor progression. However, the relationship between HIF1A-AS2 and kidney carcinoma has been rarely reported.

Studies have reported a mutual function between lncRNAs and miRNAs^50–56^, which was further confirmed by our report. LncRNAs act as miRNAs sponges or baits to titrate miRNA concentrations, thereby decreasing and preventing miRNAs from binding to specific mRNAs. Studies have also reported the roles of HIF1A-AS2 in tumors, such as HIF1A-AS2 targeting miR-548c-3p in breast cancer^31^, sponging miR-129-5p in colorectal cancer and miR-33b-5p in osteosarcoma^37,41^, sponging miR-665 in osteogenic differentiation^38^, and sponging miR-153-3p in human umbilical vein cells^56^. Our findings were consistent with these existing reports.

The miRNAs are 18–25 nucleotides of non-coding RNAs, which are involved in tumorigenesis and progression of various diseases^50–55^. Based on our knowledge, miRNA plays an indirect role in protein regulation, by binding to the 3′-UTR of specific mRNAs, to induce degradation or transcriptional suppression of target genes. The miR-30a-5p was confirmed by bioinformatics analyses and was further verified by in the present study. MiR-30a-5p has been studied in numerous diseases, such as cholangiocarcinoma^42^, osteoarthritis^43^, lung cancer^44–46^, and colorectal cancer^47,48^. Furthermore, miR-30a-5p interacts with several ncRNAs, such as LINC00461^43,44^, NORAD^45^, DLEU2^46^, LIN28B^47^, and FEZF1-AS1^48^. Based on experimental results, miR-30a-5p as a negative regulatory agent participated in the progression of renal carcinoma. The present study showed that miR-30a-5p inhibited the progression and mediated the function of HIF1A-AS2 in renal carcinomas.

*SOX4*, the target gene of miR-30a-5p, was predicted by bioinformatics analyses in addition to our experimental studies. SOX4 belongs to the SOX transcription factor family, and binds to the A/TA/TCAAG motif to regulate target gene transcriptional activity via the high mobility group domain, which regulates various biological functions, such as embryonic development and cell progression^51–55^. It regulates cell differentiation, proliferation, and metastasis. SOX4 via the lnc01694/miR-340-5p/Sox4 axis regulates gallbladder cancer^51^. SOX4 is regulated by SNHGR-miR-489-3p competitive binding in acute myeloid leukemia^52^. Sox4 is targeted by lnc-NNT-AS1/miR-142-5p axis in gastric cancer^53^, and lnc-FTX/miR-214-5p axis in the osteosarcoma^54^. SOX4 also participates in the epithelial-mesenchymal transition via lncRNA HCP5/miR-140-5p sponging^55^. Our results further elucidated the role of HIF1A-AS2 in renal carcinoma, and expanded the knowledge of the role of HIF1A-AS2 in diverse diseases.

A correlation with miR-30a-5p and HIF1A-AS2 in renal carcinomas remains unknown. Through bioinformatics analyses, we identified a putative binding site between HIF1A-AS2 and miR-30a-5p, and found that HIF1A-AS2 acted as the sponge of miR-30a-5p, which directly bound to miR-30a-5p. Furthermore, HIF1A-AS2 overexpression led to downregulation of miR-30a-5p, and HIF1A-AS2 knockdown upregulated the relative expression level of miR-30a-5p. The MiR-30a-5p inhibitor partially reversed the effects, and miR-30a-5p mimics enhanced the effects induced by HIF1A-AS2 knockdown on renal carcinoma cells. The miR-30a-5p directly targeted SOX4 to reduce the relative protein expression levels of SOX4 in renal carcinomas. Likewise, the relative protein expression level of SOX4 was upregulated during HIF1A-AS2 overexpression, to mediate the function of HIF1A-AS2. Overexpression of HIF1A-AS2 reduced the relative expression of miR-30a-5p and subsequently increased the relative protein expression of SOX4 at the post-transcriptional level in renal cancer cells. Silencing of SOX4 reversed the malignant renal carcinoma cell phenotype promoted by overexpressed HIF1A-AS2.

We elucidated the mutual function of HIF1A-AS2 and miR-30a-5p in renal carcinomas, which could provide a novel biomarker and therapeutic target for the diagnosis and treatment of renal carcinomas. We also demonstrated that the relative expression of HIF1A-AS2 was significantly increased in renal carcinoma samples and cell lines. The relative expression of HIF1A-AS2 was positively correlated with differentiation and the TNM stage in renal carcinomas. Downregulated expression of HIF1A-AS2 inhibited renal carcinoma cell proliferation or migration, and upregulated apoptosis. Overexpression of HIF1A-AS2 showed the opposite effect. We have demonstrated that HIF1A-AS2 enhanced the development and progression of renal cancers by promoting cell proliferation, migration, and reducing apoptosis. Furthermore, HIF1A-AS2 sponged miR-30a-5p in a ceRNA-dependent manner. Mechanistically, upregulation of HIF1A-AS2 decreased the relative expression of miR-30a-5p and subsequently promoted the relative expression of SOX4 and WNT signaling at the posttranscriptional level. In summary, our study showed that HIF1A-AS2 acted as a tumor promoter by miRNA sponging, and may therefore be a potential therapeutic target in renal carcinomas.

## Conclusions

The results showed that HIF1A-AS2 sponged miR-30a-5p to closely regulate SOX4 expression, and subsequently accelerated the malignant phenotypes of renal carcinoma cells and Wnt/β-catenin signaling, acting as an oncogene in the pathogenic mechanism of kidney carcinomas. Our results provide useful pathways to further explore the pathogenesis of renal carcinoma progression and development. In conclusion, the results showed that the HIF1A-AS2-miR-30a-5p-SOX4 axis played significant roles in the progression and development of renal carcinomas. HIF1A-AS2 and miR-30a-5p were the novel and important tumor biomarkers, which could be used as diagnostic biomarkers and remedial targets for malignant renal carcinomas.
