# Supplementary material for: LncRNA HIF1A-AS2 accelerates malignant phenotypes of renal carcinoma by modulating miR-30a-5p/SOX4 axis as a ceRNA
Source: Cancer Biol Med. 2021 Jun 15;18(2):587–603. doi: 10.20892/j.issn.2095-3941.2020.0209 (PMC8185866; doi:10.20892/j.issn.2095-3941.2020.0209)
Supplement: Supplementary file 1 [file cbm-18-587-s001.pdf]

Supplementary materials

Table S1 The primers for real-time qPCR

Gene	Forward or Reverse	Primer sequence
HIF1A-AS2	Forward	5′-TCTGTGGCTCAGTTCCTTTTGT-3′
	Reverse	5′-ATGTAGGAAGTGCCAGAGCC-3′
GAPDH	Forward	5′-CGCTCTCTGCTCCTCCTGTTC-3′
	Reverse	5′-ATCCGTTGACTCCGACCTTCAC-3
miR-30a-5p	Forward	5′-AACGAGACGACGACAGAC-3′
	Reverse	5′-GTAAACATCCTCGACTGGAAG-3′
SOX4	Forward	5′-AGCGACAAGATCCCTTTCATTC-3′
	Reverse	5′-CGTTGCCGGACTTCACCTT-3′

Table S2 Antibodies used for Western blot

Antibody	Description	Dilution	Supplier	Catalog	Country
SOX4	Mouse monoclonal	1:1000	Santa Cruz	sc-130633	USA
Anti-VEGF	Mouse monoclonal	1:1000	Santa Cruz	sc-7269	USA
Anti-Met	Mouse monoclonal	1:1000	Santa Cruz	sc-8057	USA
Anti-β-catenin	Mouse monoclonal	1:1000	Santa Cruz	sc-7963	USA
Anti-c-myc	Mouse monoclonal	1:500	Santa Cruz	sc-40	USA
Anti-CyclinD1	Rabbit monoclonal	1:1000	Beyotime	AF1183	China
Anti-Fra-1	Rabbit polyclonal	1:500	BBI	D260339	China
Anti-β-Actin	Mouse monoclonal	1:1000	Beyotime	AF0003	China

VEGF, vascular endothelial growth factor; Met, MET proto-oncogene, receptor tyrosine kinase; β-catenin: catenin beta 1; Fra-1, FOS like 1, AP-1 transcription factor subunit; c-myc, MYC proto-oncogene, bHLH transcription factor. SOX4, SRY (sex determining region Y)-box 4.
